# Characteristics and Injury Patterns in Electric-Scooter Related Accidents—A Prospective Two-Center Report from Germany

**DOI:** 10.3390/jcm9051569

**Published:** 2020-05-22

**Authors:** Philipp Störmann, Alexander Klug, Christoph Nau, René D. Verboket, Max Leiblein, Daniel Müller, Uwe Schweigkofler, Reinhard Hoffmann, Ingo Marzi, Thomas Lustenberger

**Affiliations:** 1 Department of Trauma, Hand and Reconstructive Surgery, University Hospital Frankfurt, 60590 Frankfurt, Germany; christoph.nau@kgu.de (C.N.); rene.verboket@kgu.de (R.D.V.); maximilian.leiblein@kgu.de (M.L.); daniel.mueller2@kgu.de (D.M.); marzi@trauma.uni-frankfurt.de (I.M.); tom.lustenberg@gmail.com (T.L.); 2 Berufsgenossenschaftliche Unfallklinik Frankfurt, 60389 Frankfurt, Germany; alexander.klug@bgu-frankfurt.de (A.K.); uwe.schweigkofler@bgu-frankfurt.de (U.S.); aerztlicher.direktor@bgu-frankfurt.de (R.H.)

**Keywords:** electric scooter, e-scooter, dislocation, traffic accident, fracture, traumatic brain injury, transportation

## Abstract

Since the introduction of rental E-scooters in Germany in mid-June 2019, the safety of this new means of transport has been the subject of extensive public debate. However, valid data on injuries and usage habits are not yet available. This retrospective two-center study included a total of 76 patients who presented to the emergency department following E-scooter-related accidents. The mean age was 34.3 ± 12.4 years and 69.7% of the patients were male. About half of the patients were admitted by ambulance (42.1%). Fractures were found in 48.6% of patients, and 27.6% required surgical treatment due to a fracture. The upper extremities were the most commonly affected body region, followed by injuries to the lower extremity and to the head and face. Only one patient had worn a helmet. In-hospital treatment was necessary for 26.3% of the cases. Patients presented to the emergency department mainly during the weekend and on-call times. This is the first report on E-scooter-related injuries in Germany. Accidents with E-scooters can cause serious injuries and, therefore, represent a further burden to emergency departments. The use of E-scooters appears to be mostly recreational, and the rate of use of protective gear is low.

## 1. Introduction

At the end of 2017, rental electrically powered scooters (E-scooters) were first introduced in the USA as a new, nationwide means of transport [1]. In Germany, approval for the use of E-scooters in public road traffic was granted on June 15, 2019. Since then, E-scooters have been distributed throughout several metropolitan regions and large cities in Germany, such as Frankfurt am Main, primarily via rental companies. In Germany, the maximum speed of the scooter is limited to 20 km/h, the rated power of the electric motor may not exceed 500 watts, and the maximum weight of the scooter is 55 kg. In addition to the existing insurance obligation, E-scooters must also be equipped with front and rear lights, as well as two separately functioning brakes. Use is permitted from a minimum age of 14 years. However, helmets are not compulsory. In Germany, the E-scooter is equivalent to a bicycle in terms of traffic law, and thus the use of sidewalks is officially prohibited. Currently, there are around 2,000 E-scooters distributed throughout Frankfurt via four major providers. According to press and police reports, traffic offences, such as the use of sidewalks, have occurred regularly since these vehicles were introduced. Given that this is a new means of transport, meaning that riders may have insufficient experience in handling the scooters, an increased number of injured persons was expected in Germany. However, reliable national data on E-scooter-related injuries are not yet available.

Likewise, worldwide data on injury patterns related to the use of E-scooters are sparse, mainly due to the short time period since their introduction to the public. In the USA, E-scooters have been approved since September 2017, and the first scientific studies in this area are now available. In these reports, the majority of injured persons were involved as scooter drivers, whereas about 10% were hit as pedestrians [2]. Approximately 40% of patients suffered craniocerebral trauma, while fractures of the extremities were the second most frequent injuries (31%) [3,4]. Despite only including a small number of cases, another American study described a relevant increase in the number of cases of E-scooter-related injury and, in particular, reports of severe craniocerebral trauma as a relevant injury [1]. These figures are in line with an observational study performed in New Zealand, where scooters have been registered since September 2018. This study also reported a relevant increase in serious injuries, particularly to the extremities, but also to the axial skeleton [5].

Despite frequent reports of accidents in the German media, there are no valid figures on injury patterns related to E-scooter accidents available to date. Likewise, the international literature is also very sparse. However, this information is important to estimate the burden these injuries pose to emergency departments and to our health care system. We hypothesize that E-scooter-related accidents result in typical high-energy injuries and that the rate of use of protective gear is low. The aim of this study was therefore to identify injury patterns following E-scooter accidents and to evaluate the need for in-hospital and surgical treatment associated with these specific injuries.

## 2. Methods

The two largest level 1 trauma centers in Frankfurt, both located within the city, participated in this retrospective analysis. The University Hospital Frankfurt is located in the south of the city, while the BG Trauma Center is located in the north of the city. Frankfurt has about 750,000 inhabitants, and due to the numerous commuters from the surrounding area, the number of people present in the city on working days is over 1,000,000. The two participating trauma centers are the largest in the city with together approximately 42,000 patient presentations in the emergency departments every year. The inclusion criteria were patients over 14 years of age who suffered E-scooter-related injuries. All patients involved in an accident with an E-scooter who presented by ambulance or independently to the emergency department of one of the two hospitals were included in this prospective observational study. Data on usage habits (e.g., wearing a helmet, using the scooter alone), injury pattern, clinical care (surgical vs. non-surgical treatment) and outcome (outpatient vs. in-hospital care) were prospectively collected, starting by the emergency department doctor during the initial treatment and after informed consent was obtained from the patient. Thereafter, the patient’s clinical course was prospectively followed, and all related data were collected by the study coordination team. Each chronical illness was registered as co-morbidity, as well as each permanent medication (except oral contraception) was registered as pre-existing medication. The primary outcome included all injuries diagnosed during the clinical course. These injuries were categorized as serious (severe traumatic brain injury, fractures) or minor (contusions, lacerations). Secondary outcomes included need for surgery, in-hospital and intensive care unit treatment. This study was approved by the local ethics committee of the Johann-Wolfgang Goethe University (EV 19/408).

### Statistical Analyses

Values are reported as mean ± standard deviation (SD) for continuous variables and as percentages for categorical variables. The *p*-values for categorical variables were derived from the chi-square or two-sided Fisher’s exact test. All analyses were performed using Statistical Package for Social Sciences software (SPSS for Mac; version 24.0; SPSS Inc., Chicago, IL, USA).

## 3. Results

Over the 9-month study period, a total of 76 patients were included. The mean age of patients was 34.28 ± 12.4 years (range 15–67 years). Two patients were younger than 18 years of age and 69.7% were male. Of the 76 patients, 13.2% (n = 10) stated that they suffered from a pre-existing morbidity, and 10.5% (n = 8) were on permanent medication of any kind prior to the accident.

In total, 70 patients (92.1%) suffered from an accident without any external influence, whereas five patients (6.6%) were admitted after a collision with a car and one patient (1.3%) collided with a forklift truck. All patients used the E-scooter alone, with a rate of first use of 32.9%. Self-admission was registered in 44 patients (57.9%), and 32 patients (42.1%) were admitted by ambulance. In 27.6% of the cases, the accident occurred on wet ground. Only one patient was using a helmet while using the E-scooter. Nine patients (11.8%) were initially unconscious; however, endotracheal intubation was not necessary in any of the patients, neither preclinically nor in-hospital. No differences with regard to usage habits, accident characteristics or preinjury medications and comorbidities were found between male and female patients (Table 1).

Table 2 presents the injury pattern. Overall, 43 patients (56.6%) suffered from at least one serious injury, with two patients (2.6%) suffering two serious injuries. A total of 21 patients (27.6%) required surgical management for their injuries. The upper extremities were the main body region affected, with a total of 36 injuries (47.4%), of which 26 were considered serious. Thirteen patients (17.1%) had to undergo surgical procedures for their upper extremity injury. The second most common injury location was the head and face (n = 29, 38.2%), followed by injuries of the lower extremities (n = 28, 36.8%). Injuries of the chest were registered in 9.2% of patients (n = 7). No abdominal injuries were found.

**Figure 1:**
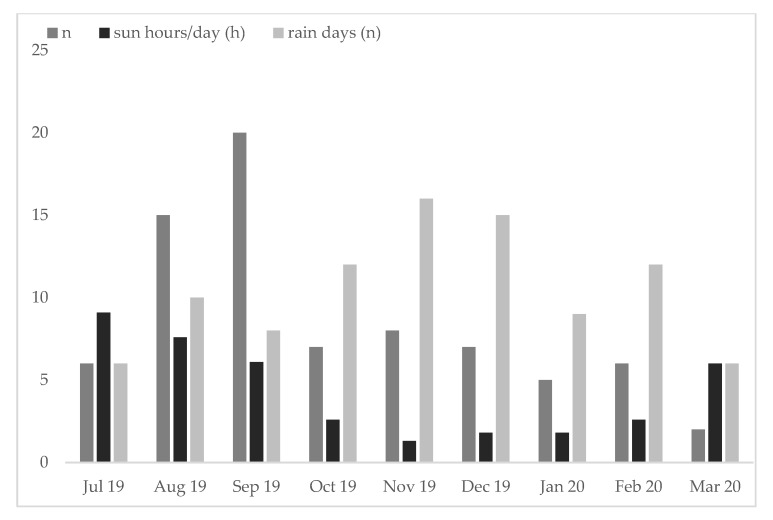
The highest number of accidents was registered during the summer months. In March 2020, it should be noted that initial restrictions were imposed by the SARS-CoV-2 pandemic. Furthermore, mean hours of sun per month as well as rain days per month are depicted.

Most accidents were registered during summer (August/September), with lower numbers observed in winter. Due to restrictions imposed during the SARS-CoV-2 pandemic, numbers for March 2020 were the lowest (Figure 1).

**Figure 2:**
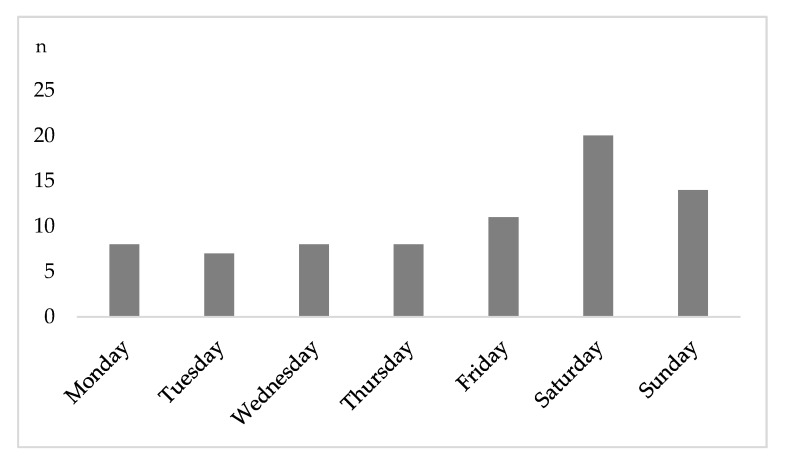
Number of presentations by day. By far the most patients were treated on Saturday.

Overall, 40.8% (n = 31) of hospital admissions occurred between Friday 4 p.m. and Monday 6 a.m. (weekend on-call time). Most injuries were recorded on Saturday (n = 20; Figure 2).

**Figure 3:**
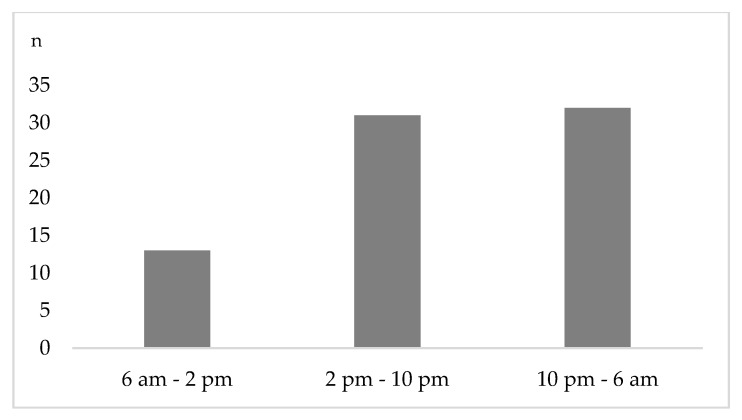
Time dependent presentation of patients after e-scooter accidents. Most patient contacts were registered during on-call time.

Figure 3 presents the time distribution of emergency department presentation. Throughout the week, 17.1% (n = 1 3) of the accidents were registered between 6 a.m. and 2 p.m., while 40.8% (n = 31) and 42.1% (n = 32) were seen between 2 p.m. and 10 p.m. and between 10 p.m. and 6 a.m., respectively (Figure 3). A significantly higher rate of head trauma was seen during on-call time (10 p.m.–6 a.m.: 0% vs. 6 a.m.–2 p.m.: 9.7% vs. 2 p.m. –10 p.m.: 31.3%, *p* = 0.02).

Overall, 73.7% of the patients (n = 56) were treated as outpatients. Of those, despite having an indication for in-hospital treatment, four patients (equal to 5.3% of all patients) discharged themselves from the hospital against medical advice. In total, 26.3% of the patients (n = 20) required in-hospital treatment with a mean length of stay of 5.1 ± 4.5 days (minimum 1 day, maximum 15 days). Of those 20 patients, four required intensive medical care. In all cases, the reason for intensive care unit admission was severe traumatic brain injury including intracerebral bleeding (n = 1), subdural hematomas (n = 2) and subarachnoidal bleeding (n = 2). The mean intensive care unit length of stay was 3.3 ± 2.2 days. No fatalities were registered.

## 4. Discussion

To the best of our knowledge, this is the first study of E-scooter-related injuries in Germany. The first worldwide introduction of rental E-scooters took place in 2017 in San Francisco, USA. Since then, public discussion focused on increasing accident numbers and the involvement of E-scooters in traffic accidents has increased [6,7,8]. However, due to the short time period since the introduction of this new means of transport, data on injury characteristics and prevention are still scarce. Only a few articles reporting E-scooter-related injury patterns and their outcomes have been published so far [9]. In a study performed in New Zealand over a 4-month period, Mayhew et al. recorded 46 patients who were admitted to a level 1 center after falling from an E-scooter [5]. A study by Trivedi et al. reported on 90 patients who presented with E-scooter-related trauma in Texas over a 7-month period. Ishmael and colleagues recently analyzed the surgical procedures that have become necessary following an E-scooter accident [4]. Comparing these publications with our results, a similar patient picture emerges from all studies. The gender distribution is comparable, with about two-thirds being male, and the patients are young to middle aged. Although E-scooter use by minors is permitted in all countries, the proportion of juvenile patients was low in all investigations.

In our study, peaks of accident-related emergency department presentations were observed at weekends and in the late evening and night hours. Similarly, in the studies by Mayhew et al. and Ishmael et al., the majority of emergency department presentations occurred in the summer months, as well as during on-call times [4,5]. This accumulation of patients outside regular working hours places further strain on the already scarce resources in emergency departments. Moreover, the observed usage characteristics support the assumption that E-scooters are more likely used as leisure equipment and not—as hoped by politicians—as an additional means of transport to a workplace or to bridge the last distance between home and the local public transport stop. In this context, a recent study from California highlighted that E-scooters are particularly popular with tourists [4].

The limited data available to date suggest that a high percentage of accidents involving E-scooters will result in serious injuries, usually to the head and extremities. This finding is further substantiated by our investigation, as we found serious injuries such as traumatic brain injury and fractures in 56.6% of patients. The severity of the head injuries varied, but all intensive care stays were nevertheless attributable to traumatic brain injury. Due to the relatively high speed on small wheels, which is comparable to that of cyclists, and the low fall height with a short reaction time, the extremities, especially the upper extremities, and head are the most commonly affected body areas [2,6,10]. As a consequence, the risk for relevant long-term functional limitations following E-scooter accidents should not be underestimated. Complex articular fractures and ligamentous injuries, in particular to the elbow joint, may result in permanent instability and a reduced range of motion [11]. The unbraked impact of the head without a protective helmet may also cause permanent disability and significant restrictions of the individual’s preinjury lifestyle. Furthermore, the high number of midface injuries and tooth fractures might ultimately lead to a cosmetically unfavorable outcome.

These significant injury patterns are not only due to the high speed and short reaction time associated with E-scooter use, as mentioned above, but also due to the very low rate of use of protective measures, such as helmets. In all available studies, including ours, the use of E-scooters without a helmet was found in almost 100% of cases. Using a helmet might probably have reduced the rate of concussions and severe traumatic brain injury, even if the face is not protected by a classic bicycle helmet. In addition, in the context of leisure use, a high degree of inexperience in handling an E-scooter has to be assumed, which further increases the risk of being involved in an accident. In this respect, the current literature demonstrates a high rate of intoxicated patients, ranging from 17.8% up to 36.6% in different studies [3,10]. Unfortunately, due to restrictions imposed by our local ethics committee, we were not able to measure the blood alcohol level of our patients. Nevertheless, from a clinical standpoint, a high percentage of patients appeared to be intoxicated to some degree at the time of presentation.

A comparison of the E-scooter accident mechanism with other sports is rather difficult due to the special combination of high speed and proximity to the ground. It is noteworthy, however, that the injury patterns appear largely similar to those observed after skateboarding, skiing and snowboarding accidents [12,13]. In all of these sport activities, the fall height is low, the speed is high, and the reaction time is short. Skateboarding accidents, for example, did not only show a high percentage of traumatic brain injuries, but also a high rate of injury to the extremities. In contrast, injuries of the chest and abdomen were more rarely found. As a result of snowboarding and skiing accidents, glenohumeral dislocations—also seen in our study—are frequently observed [14]. The treating emergency department teams should therefore be aware of these injury patterns, which are otherwise only known from high speed or extreme sports. Furthermore, similar injury patterns have also been described for accidents involving hoverboards, which are another newer means of transport. Here, the use of helmets and wrist guards have been strongly recommended [15]. These safety measures could similarly reduce the number of significant injuries after E-scooter accidents. Both in our study and in the available literature on E-scooter-related accidents to date, the use of helmets is virtually non-existent. In the present study, only a single injured person was wearing a helmet, even though the benefit of helmets in preventing traumatic brain injury has been well analyzed and proven in the past [16,17,18]. Considering the rate of head injuries following E-scooter accidents, the use of a helmet should therefore be strongly recommended. Additionally, adapted protective equipment may be needed to protect the face and extremities.

### Limitations

As only level 1 centers took part in the present study, there was incomplete coverage of the city, as patients with minor injuries may have independently visited level 2 and 3 centers in the city. It can be assumed, however, that patients with multiple and/or serious injuries are primarily assigned to one of the maximum care centers. The data presented are also influenced by population density, topography of the city, the public transport system and other parameters, which should be taken into account when comparing the data with future studies. Due to the short investigation period, the present study provides only a first overview of the injury patterns that should be expected and the burden that E-scooter-related injuries pose for emergency departments. For example, in the current literature, no fatalities have been documented so far, although these have to be expected in the case of further use in road traffic. In the future, multicenter studies should be carried out to evaluate the injury patterns and outcomes more precisely.

## 5. Conclusions

Electric scooter-related accidents are associated with a significant number of serious injuries. These injuries include fractures and lacerations of the midface, as well as fractures and dislocations of the upper extremities, which often require surgical treatment. In light of the significant rate of severe injuries, the use of protective clothing, especially helmets, is strongly recommended.

## Figures and Tables

**Table 1:** Basic data, comorbidities, and need for surgical treatment following E-scooter accidents stratified by gender.

	Total (%) n = 76	Male (%) n = 53	Female (%) n = 23	*p* Value
Hospitalization by ambulance, % (n)	42.1% (32)	41.5% (22)	43.5% (10)	0.81
Self-inflicted accident, % (n)	92.1% (70)	92.5% (49)	91.3% (21)	1
First use, % (n)	32.9% (25)	32.1% (17)	34.8% (8)	1
Wet ground, % (n)	27.6% (21)	34% (18)	17.4% (4)	0.29
Helmet worn, % (n)	1.3% (1)	1.9% (1)	0%	1
Co-morbidities, % (n)	13.2% (10)	17% (9)	4.3% (1)	0.43
Pre-existing medication, % (n)	10.5% (8)	13.2% (7)	4.3% (1)	0.66
Unconsciousness, % (n)	11.8% (9)	11.3% (6)	13% (3)	1
Outpatient care, % (n)	73.7% (56)	75.5% (40)	69.9% (16)	0.58
Surgical treatment, % (n)	27.6% (21)	30.2% (16)	21.7% (5)	0.58

**Table 2:** Distribution of injuries and required surgical treatment in all patients with E-scooter-related accidents (n = 76).

Body area	Injury	n (%)	Surgery n (%)
**Head**	**Total**	**13 (17.1%)**	**0**
	intracerebral bleeding	1	
	subarachnoidal hemorrhage	2	
	subdural hemorrhage	2	
	concussion	3	
	minor injury	5	
**Face**	**Total**	**16 (21.1%)**	**4 (5.3%)**
	midface fracture	6	4
	laceration	4	-
	tooth fracture	4 **	-
	minor injury	2	-
**Chest**	**Total**	**7 (9.2%)**	**0**
	serial rib fractures	1	
	minor injury	6	
**Abdomen**	**Total**	**0**	**0**
**Upper extremity**	**Total**	**36 (47.4%)**	**13 (17.1%)**
	fractures	22 (28.9%)	12 (15.8%) *
	shoulder	7	5
	elbow	7	3
	forearm/wrist/hand	8	4
	dislocations	3 (3.9%)	1 (1.3%) ^#^
	shoulder	2	-
	elbow	1	1
	minor injury	10 (13.2%)	0
**Lower extremity**	**Total**	**28 (36.8%)**	**5 (6.6%)**
	fractures	7 (9.2%)	6 (7.9%) *
	pelvis	1	-
	knee	2	2
	ankle	3	3
	foot	1	1
	minor injury	21 (27.6%)	0

Some patients suffered from multiple injuries. Minor injuries included contusions and lacerations. * Fractures required open or closed reduction and internal fixation, ^#^ Dislocations required reconstruction of ligamentous injuries, ** Treatment by dentist.
